# Gender differences in research performance and its impact on careers: a longitudinal case study

**DOI:** 10.1007/s11192-015-1775-3

**Published:** 2015-11-12

**Authors:** Peter van den Besselaar, Ulf Sandström

**Affiliations:** Department of Organization Sciences & Network Institute, Vrije Universiteit Amsterdam, Amsterdam, The Netherlands; INDEK, KTH Royal Institute of Technology, Lindstedtsv. 30, 10044 Stockholm, Sweden; Örebro University, Örebro, Sweden

**Keywords:** Gender bias, Academic careers, Performance differences, Longitudinal study

## Abstract

We take up the issue of performance differences between male and female researchers, and investigate the *change* of performance differences during the early career. In a previous paper it was shown that among starting researchers gendered performance differences seem small to non-existent (Van Arensbergen et al. 2012). If the differences do not occur in the early career anymore, they may emerge in a later period, or may remain absent. In this paper we use the same sample of male and female researchers, but now compare performance levels about 10 years later. We use various performance indicators: full/fractional counted productivity, citation impact, and relative citation impact in terms of the share of papers in the top 10 % highly cited papers. After the 10 years period, productivity of male researchers has grown faster than of female researcher, but the field normalized (relative) citation impact indicators of male and female researchers remain about equal. Furthermore, performance data do explain to a certain extent why male careers in our sample develop much faster than female researchers’ careers; but controlling for performance differences, we find that gender is an important determinant too. Consequently, the process of hiring academic staff still remains biased.

## Introduction

The research literature has shown the performance gaps between male and female researchers since long been indicated, with men on average publishing more papers, and receiving more citations than female researchers (Cole and Zuckerman, 1984; Long, 1992; Xie and Shauman, 1998; Nakhaie, 2002; Prpic, 2002; Penas and Willett, 2006; Symonds, et al., 2006; Taylor, et al., 2006; Ledin, et al., 2007; Abramo, et al., 2009). However, with regard to citations per publication some studies claim that no gender differences exist (Penas and Willett, 2006; Ledin, et al., 2007; Tower, et al., 2007). A few studies even found a higher citation score for women than for men (Long, 1992; Powell, et al., 2009; Sandström, 2009a, b). Nevertheless, on average, total impact of female researchers used to remain lower than of male researchers, due to the lower productivity. This productivity difference emerges already in the early career and then does not disappear (Symonds, et al., 2006).

Why is this productivity difference important? As science claims to be a *meritocracy*, gender related variation in performance could (and should!) explain gender related differences in grant decisions, and gender related differences in academic careers. But if differences in career success and in grant success do not relate to differences in performance, we would have typical examples of *gender bias* in contrast to the claimed meritocracy.

### Gender bias in science

That bias may play a role was very clearly put on the agenda when Wennerås and Wold (1997) published their results of a study on grant decision-making. They showed that not meritocracy was the standard, but cronyism: having friends in the relevant committees proved to help considerably in getting a grant. On top of that, they also showed the role of gender bias: women needed a substantially (160 per cent) higher performance than male researchers to be successful in biomedical grant applications. Replicating that study some 10 years later, Sandström and Hällsten (2008) found again nepotism, but no sexism anymore: female researchers even had a slightly better chance than male researchers. Obviously, the council studied in both papers changed its gender policy in the meantime.^1^ After the Wennerås and Wold study, quite a few other studies found that that gender matters, but some evidence suggests differently in different disciplines. For example, a Dutch study showed that in science fields female researchers received positive evaluations and high success rates, even higher than could be expected from past performance. In contrast, the life sciences were characterized by negative gender bias, as no substantial differences between track records of unsuccessful women and successful men were found (Brouns 2000).

Apart from the issue of gender bias in grant reviewing, there is also an issue about gender bias in academic recruitment and selection, leading to lower success rates of female applicants (Van den Brink et al. 2006; Van den Brink 2009). Female researchers have a slower career, and on average end at lower positions; women are still underrepresented in the higher academic positions and men outnumber women in positions of formal power, authority and high income (Xie and Shauman 1998; Timmers et al. 2010). Here again the question is at stake as whether these career differences relate to performance differences, or are based on biased decision-making.

In a recent review, Ceci and Williams (2011) discuss the evidence about gender bias in science, in journal reviewing, grant funding, and in hiring. They claim not to find evidence supporting the existence of discrimination against women in science. The authors conclude that the unequal position of women in science would be based on *quality differences* between male and female researchers that may partly be based on *own career related choices*, and partly on *discriminatory arrangements* not in science but *in society at large* — e.g., inequalities related to division of domestic work and child care. If this analysis is correct, we are back from gender bias to performance differences.

The main problem with most studies arguing that no gender bias exists is that they do not take performance into account at all. This is also the case with recent meta-reviews (Mutz et al. 2014; Marsh et al. 2009), claiming that gender bias no longer exists in peer review. These studies do not refer to performance, but look only at the success rates.

This is remarkable, as bias can only be measured against performance differences. More directly, if the slower career and the glass ceiling would be determined by *performance differences alone*, one might be willing to accept this—and focus policy on improving female performance. But with this omission, the studies mentioned actually are not very instructive and fail to show what they claim. They have no information about whether the applicants are statistically representative of their respective groups (male–female), and they also lack information about any self-selection processes that correlate with performance. For example, it might be that self-selection of female applicants applying for a job is stronger than of male researchers in the sense that female applicants have a higher average performance than male applicants. If that is the case we would expect better average bibliometric scores for the female group and, on average, less good results for the male group. These performance data then could be correlated with the committee decisions. We assume that bibliometric data are more or less unbiased for each applicant *group* and that full bibliometric data with relative citations scores will produce relevant correlates to the grading and ranking procedures of standing and ad hoc committees.

### Productivity differences

Several explanations of the *productivity puzzle* (Cole and Zuckerman 1984) have been proposed: scientific ability, self-selection, social selection, and accumulated disadvantage (Zuckerman 2001).^2^ According to the scientific ability explanation, male and female academics differ in biological and psychological characteristics, influencing research output. However, more recent research did not find a direct gender ability effect as was established in earlier research (e.g. Xie and Shauman 1998). Above that, one can observe at all levels of education that girls tend to outperform boys, which also contradicts this assumption (Buchmann et al. 2008; Pekkarinen 2008).

The second explanation is more widespread. In the early career, researchers get children and start a family. In fact, this may affect women more than men, as women still to the larger part of domestic work and child-care. This may translate in less time for research and therefore a lower scientific performance in the early career (Long 1992; Symonds et al. 2006). Only later in their career women more or less catch up with male researchers (Long 1992; Symonds et al. 2006), but this lower early productivity has a negative effect on careers (Prozesky 2008; Fuchs et al. 2001; Hunter and Leahey 2010; Karamessini 2004).^3^ However, other studies indicate that the academic career of female scientists does not suffer from parenthood (Fox & Faver 1985; Cole & Zuckerman 1991; Astin & Bayer 1979; Dryler 2011).

One may argue that getting kids is choice, but the fact that the bulk of domestic labor and of child-care is done by women, is no ‘self-selection’ but a social selection process (c.f. Fox 2005, Fox et al. 2011). This brings us to a variety of differences between male and female researchers, which are partly choice (self-selection) and partly based on social selection. Female researchers have a lower degree of specialization (Leahey 2006), tend to work in other disciplines, tend to focus more on teaching, tend to work at universities and departments with lower reputation, and have a less developed international collaboration and co-authoring network (Allison and Long 1990; McNamee et al. 1990; Dundar and Lewis 1998; Prpic 2002; Lee and Bozeman 2005; Bland et al. 2006; Carayol and Matt 2006; Leahey 2006; Taylor et al., 2006; Puuska 2010), which all affects performance and career in a negative way. However, Badar et al., (2013) report the opposite, but in the context of a developing country. This suggests that a specific study on gender and performance focusing on the latter context may be useful. Furthermore, women receive less academic support and mentoring than men (Landino and Owen 1988; Fuchs et al. 2001). This may be a disadvantage for women too, as academic careers depend on support by academic mentors (Van Balen et al. 2012). A slower career progress recursively may lead to less research and more teaching (Taylor et al. 2006; Snell et al. 2009), and consequently to less scholarly productivity. The accumulation of all these self and social selections over time leads to *cumulative disadvantage* (Zuckerman 2001).

However, over time gender roles and responsibilities in family life are changing (Xie and Shauman 1998; Taylor et al., 2006; Prozesky 2008), which may also influence work and career orientation of women. One may expect that the gradually changing gender roles in the last decades in many countries may also result into changed behavior. Indeed we have witnessed increased performance of girls in the educational system, and at all levels, girls are outperforming boys (Buchmann et al. 2008; Pekkarinen 2008). Recent data suggest that the performance gap has been closed for PhD students (Miller & Wai 2015; Ceci et al. 2014). If this is indeed a slow and generational process, one would wonder whether this *gender performance turn* has also reached the research system, first of all the generation of early career researchers.^4^

An earlier study (Van Arensbergen et al. 2012) indeed confirmed (i) the traditional gendered performance pattern for the older generations of researchers, but (ii) when isolating the group of *early career researchers*, gender differences have become smaller (in e.g., economics) or are disappearing (in psychology). The question remains as whether women now at a later moment face the performance drop.

## Research questions

So our *first research question* is: Do gender differences in research performance emerge at a later age? The general question behind this is whether we are observing a change in the *academic life cycle* of female researchers (the productivity dip emerges now later than in the past), or whether is it a *generational change* meaning that the productivity gap has disappeared in the younger generations.

Around 2004, the young male and female researchers in our sample not only had about the same average performance, but also had about the same position: they were all <3 years after their PhD and generally at the postdoc level. This makes the sample useful for studying career development, which in a meritocracy is expected to go hand in hand with performance development. Therefore our *second research question* is: Have gender career differences occurred, and, if so, can they be explained by performance differences? With this study we hope to improve our understanding of gender differences in science, by *taking performance differences into account*—something that is lacking from many former studies.

## Data and method

Our sample consists of some 400 researchers that have submitted proposals to an early career grant program of a social science council in the Netherlands between 2003 and 2005. As applying for (such) grants is considered an essential activity during the early research career, it can be assumed that the data cover the community of young social science researchers in the Netherlands during that period rather well.

From the total set of young researchers, we selected here the young researchers in psychology, in behavioral & educational research, and in economics. This selection was made because for these fields the Web of Science is covering academic output relatively well, enabling us to use a bibliometric approach to performance measurement. *Relatively well* does not mean that all output is covered; here it means that the WoS indexed journals are considered as the most important publication venues, within relevant communities such as the faculties involved and the research council (Van den Besselaar & Leydesdorff 2009).^5^

This selection resulted in a set of 262 researchers, of which 19 are deleted because of missing data—we could not trace them anymore. This leaves us 104 early career economics researchers (73 % male), 48 behavior and education researchers (38 % male), and 91 psychology researchers (44 % male). On average 45 %, of the researchers in the sample are female. The researchers are in our sample as they applied for an early career grant in 2003 (24 %), 2004 (38 %), or 2005 (38 %).

Homepage, CV and Web of Science provide us with the following information about academic performance and career:

- An overview of their *publications*—which was used to validate the performance data as downloaded from the WoS.
- The academic *position* in 2003–2005,^6^ which (in most cases) was postdoctoral researcher;
- The *current position* (early 2014) is measured on an ordinal scale with values from 10 (teacher) to 16 (full professor); this are the codes used for the positions in universities’ job structure. About 20.6 % of the cases was early 2014 full professor (score = 16), 28.3 % was associate professor (score = 14), 27.5 % assistant professor (score = 12), 2.1 % senior researcher (score = 11), 14.2 % researcher (score = 11), and 3 % of the applicants was in teaching positions (score = 10). The remaining 4.3 % has a career outside academia. The small number of applicants that went to positions outside academia is not included in the analysis, as it is difficult to integrate their positions into the academic rank system. Furthermore, those that left academia stopped publishing, so we do not have the scores on the independent variables.
- In between *affiliations*;
- The level of *mobility*: 47 % of the sample showed no mobility, 32 % showed national mobility, and 21 % showed international mobility.

In order to make an adequate bibliometric dataset, we retrieved publication data from the Web of Science (*SCI*-*expanded, Social Science Citation Index; Arts and Humanities Citation Index)* using the following query:

- AU = last_name first–initial* AND (CU = Netherlands OR CU = *country name*) AND DT = (article OR letter OR note OR proceedings paper OR review) and PY = 2001–2012

‘Country name’ in the query refers to countries where the researcher has worked according to his or her CV. The data were manually cleaned, by comparing the found WoS records with the publication lists found on the Web. In this way we could delete papers that were authored by others with the same name. In cases where we missed paper from the publication list, we searched for the missing titles in WoS, and added it to the set. Generally, missing papers was due to the fact that authors used different initials. As well known, disambiguation and entity resolution are time consuming. But it creates a reliable data set, which is needed given sample size involved.^7^ With the BMX tool (Sandström & Sandström 2009) the following field normalized citation indicators were calculated:

- P: Number of publications, full counting
- Frac P: Number of publications, fractional counting based on author shares
- NCSf: Field normalized citation score, until 2014
- NCSf2y: Field normalized citation score, 2 years window
- TOP x %: Share of publications in the set of (1, 5, 10, 25 and 50 %) highest cited publications, field normalized

The sample consists of three groups applying for a career grant in 2003, 2004, and 2005 respectively. Publications were included from 3 years before the grant (including the application year) until 2012. Citations were measured in two ways: (i) With a time window of 3 years (including the publication year). So for a paper published in 2005, citations are counted until 2007. (ii) Without a time window, which of course means that the early publications (in each period) have higher chance to become cited, than more recent papers. We include citations until the end of 2014. Of course, citations as such say something about overall impact, but not about the real important scientific contributions. For identifying those, we use the share of publications of a researcher in the set of highest cited publications. We use several classes of ‘top papers’: the top 1 % highest cited papers; the top 5 % highest cited papers; the top 10 % highest cited papers; the top 25 % highest cited papers; the top 50 % highest cited papers. All citation-based indicators are size-independent and field normalized. Obviously these different measures are influenced by the number of years, a researcher is active. Therefore we include the application year (reflecting the academic age) as confounding variable.

As the data are not normally distributed, but rather skewed, we use of non-parametric statistics. We compare means, medians, and the distributions of male and female performance. Comparison is done using the SPSS22 procedures *non*-*parametric tests* and *Anova*. Apart from testing bivariate gender differences, we also test a multivariate model predicting career level, using the following independent variables: performance (publications; field normalized citation score), academic age (see note 7), level of mobility, discipline, and gender. As the dependent variable is ordinal, we use of *Generalized Linear Models* the ordinal response (multinomial) model with a cumulative logit link function.

## Findings

### Early career phase

In (Van Arensbergen et al. 2012), it was shown that the gendered performance differences seem to be disappearing in the youngest generation of researchers. The analysis was based on publications and citations, and differentiating between social science disciplines. However, no field–normalized indicators were used. We now first test whether the revealed pattern remains when using the field-normalized indicators. As Table 1 shows, the differences are small. In economics, the scores of the male applicants are generally somewhat higher than of females, in behavioral and education studies and in psychology it is the other way round.

**Table 1:** Past performance of male and female early career researchers

		P	Frac P	NCS^b^	NCS2y^b^	TOP1%^b^	TOP5%^b^	TOP10%^b^	TOP25%^b^	TOP50%^b^
*All*										
*M*	Mean	2.63	0.97	0.91	0.84	0.01	0.06	0.11	0.22	0.42
*N* = 134	Median	2.00	0.79	0.48	0.42	0.00	0.00	0.00	0.00	0.40
*F*	Mean	2.41	0.82	1.07	0.88	0.01	0.06	0.11	0.29	0.50
*N* = 111	Median	2.00	0.60	0.83	0.54	0.00	0.00	0.00	0.00	0.50
*Economics*										
*M*	Mean	1.59	0.72	0.81	0.71	0.00	0.07	0.11	0.29	0.35
*N* = 76	Median	1.00	0.50	0.31	0.11	0.00	0.00	0.00	0.00	0.06
*F*	Mean	1.24	0.70	0.65	0.40	0.01	0.03	0.05	0.15	0.29
*N* = 29	Median	1.00	0.50	0.07	0.00	0.00	0.00	0.00	0.00	0.00
*Behavioral and educational science*										
*M*	Mean	5.44	1.70	1.10	1.01	0.01	0.09	0.11	0.23	0.39
*N* = 18	Median	4.00	1.22	0.47	0.74	0.00	0.00	0.00	0.06	0.26
*F*	Mean	3.03	0.87	1.10	1.03	0.02	0.05	0.10	0.33	0.53
*N* = 31	Median	2.00	0.67	0.86	0.58	0.00	0.00	0.00	0.17	0.57
*Psychology*										
*M*	Mean	3.35	1.13	1.02	0.99	0.01	0.05	0.10	0.25	0.56
*N* = 40	Median	3.00	0.79	0.83	0.77	0.00	0.00	0.00	0.19	0.61
*F*	Mean	2.76	0.88	1.30	1.08	0.01	0.09	0.16	0.34	0.61
*N* = 50	Median	2.00	0.67	1.16	0.86	0.00	0.00	0.00	0.33	0.78

Cohort early career grant applicants 2003–2005—psychology, economics, behavioral and education studies

^a^Citations based indicators are field-normalized

In Table 2, we test whether the small differences in medians and distributions are statistically significant. For none of the performance variables this is the case if we use *all applicants*. Also when distinguishing between the three *fields*, none of the differences between medians is statistically significant at 0.05, and only one at 0.10. Only a few mean rank distributions differ significantly: In economics male researchers have significantly more papers in the top 1 % cited class, but the other differences are not significant. In behavioral and education research, men publish significantly more than women—in this case, the difference between the medians is also significant at 0.10. In psychology, none of the differences are statistically significant. In other words, the here deployed field normalized indicators confirm the earlier findings (in Van Arensbergen et al. 2012) about disappearing gendered performance differences in the youngest generation researchers. The main difference in relation to the earlier contribution by Van Arensbergen et al. (2012) is that they found that women tend to outperform men in the top of the distribution. The new data and analysis suggest equal performance.

**Table 2:** Gendered performance differences—early-career researchers

	P	Frac P	NCS	NCS2y	TOP1%	TOP5%	TOP10%	TOP25%	TOP50%
*All*									
Mann–Whitney *U*	7251	7043.5	6851	7262	7187.5	7080.5	6964	6760.5	6668
Wilcoxon *W*	13,467	13,259.5	15,896	16,307	16,232.5	16,125.5	16,009	15,805.5	15,713
*Z*	−0.343	−0.721	−1.073	−0.326	−1.259	−0.965	−1.085	−1.35	−1.445
Sign. distribution	0.732	0.471	0.283	0.744	0.208	0.335	0.278	0.177	0.149
Sign. median	0.885	0.220	0.278	0.753	0.347	0.391	0.290	0.356	0.278
*Economics*									
Mann–Whitney *U*	962.5	1031.5	960	877	1026	1046.5	1065.5	996.5	1016
Wilcoxon *W*	1397.5	1466.5	1395	1312	3952	1481.5	1500.5	1431.5	1451
*Z*	−1.038	−0.520	−1.049	−1.768	−2.300	−0.748	−0.418	−0.961	−0.669
Sign. distribution	0.299	0.603	0.294	0.077	0.021	0.454	0.676	0.337	0.504
Sign. median	0.773	0.833	0.416	0.127	0.130	0.701	0.961	0.461	0.567
*Behavioral and educational science*									
Mann–Whitney *U*	185.5	181.5	272	267	276	256	272	237.5	222.5
Wilcoxon *W*	681.5	677.5	443	763	772	752	768	408.5	393.5
*Z*	−1.960	−2.031	−0.146	−0.253	−0.118	−0.598	−0.168	−0.903	−1.200
Sign. distribution	0.050	0.042	0.884	0.801	0.906	0.550	0.867	0.367	0.230
Sign. median	0.095	0.112	0.435	0.685	0.742	0.815	0.945	0.851	0.851
*Psychology*									
Mann–Whitney *U*	922.5	915	883	917	992	880	908.5	866	909
Wilcoxon *W*	2197.5	2190	1703	1737	2267	1700	1728.5	1686	1729
*Z*	−0.636	−0.693	−0.953	−0.677	−0.182	−1.366	−0.853	−1.132	−0.760
Sign. distribution	0.525	0.488	0.340	0.498	0.856	0.172	0.394	0.257	0.448
Sign. median	0.832	0.525	0.138	0.832	0.775	0.312	0.789	0.289	0.138

See notes Table 1

### Mid career phase

How did these researchers develop in the about 10 years after the previous measurement? In that period, various gendered mechanisms may have worked, as suggested in the literature. For example, male and female researchers may have faced different family responsibilities, or different career paths. As the average age of the group is about 43, this should be visible in the collected performance data. Therefore we test whether performance differences between men and women have increased between 2004 and 2014, in a for men favorable direction. In Table 3, we show mean and median scores of male and female researchers.

**Table 3:** Post performance of male and female researchers, 9 years later

		P	Frac P	NCS	NCS2y	TOP1%	TOP5%	TOP10%	TOP25%	TOP50%
*All*										
*M*	Mean	16.0	5.6	1.31	1.23	1.49	7.03	14.4	31.0	58.4
*N* = 128	Median	10.5	4.5	1.11	1.06	0.00	0.8	9.93	29.0	59.8
*F*	Mean	13.66	3.8	1.29	1.18	1.54	5.83	13.5	35.0	62.4
*N* = 105	Median	8.00	2. 7	1.11	1.17	0.00	0	5.86	35.0	63.3
*Economics*										
*M*	Mean	8.82	3.91	1.28	1.20	1.37	6.31	14.2	29.0	56.4
*N* = 71	Median	8.00	3.50	0.98	0.91	0.00	0.00	6.33	26.0	56.3
*F*	Mean	5.81	2.59	1.28	1.11	1.72	5.94	14.0	34.0	59.5
*N* = 27	Median	5.00	2.33	1.09	1.22	0.00	0.00	0.00	35.0	56.4
*Behavioral and educational science*										
*M*	Mean	23.2	7.00	1.25	1.20	1.45	6.58	12.4	32.0	59.6
*N* = 17	Median	21.0	7.76	1.18	1.11	0.00	5.05	11.1	32.0	58.7
*F*	Mean	16.9	3.88	1.30	1.24	0.94	6.26	12.7	35.0	64.5
*N* = 28	Median	9.0	3.26	1.37	1.32	0.00	0.10	7.67	35.0	69. 5
*Psychology*										
*M*	Mean	25.6	7.88	1.40	1.31	1.71	8.49	15. 8	35.0	61.6
*N* = 40	Median	21.0	7.73	1.23	1.24	0.00	4.28	11.8	36.0	67.0
*F*	Mean	16.1	4.34	1.29	1.19	1.77	5.54	13.7	35.0	62. 8
*N* = 50	Median	10.5	3.00	1.08	1.09	0.00	1.09	8.66	34.0	63.0

See notes Table 1

In publications, we see a difference in favor of men in all the fields, so productivity of male researchers has developed stronger; in citations the differences are rather small, sometimes in favor of male researchers, sometimes in favor of female. Do we have to reject the null-hypothesis that men and women perform equally well? In order to test this, we use a Mann–Whitney test. With this we can test whether the performance distributions for male and female researchers are different, and whether the medians are different. The results are in Table 4.

**Table 4:** Gendered performance differences—mid-career researchers

	*P*	Frac P	NCS	NCS3y	TOP1%	TOP5%	TOP10%	TOP25%	TOP50%
*All*									
Mean rank (male)	126.7	133.1	115.8	115.0	119.8	120.9	120.7	112.4	112.3
Mean rank (female)	105.2	97.4	119.4	119.8	113.6	112.3	112.3	121.4	122.8
Mann–Whitney *U*	5476	4666	6566	6469	6363	6226	6229	6148.5	6113
Wilcoxon *W*	11,041	10,231	14,822	14,725	11,928	11,791	11,794	14,277	14,369
*Z*	−2.432	−4.012	−.301	−.490	−.950	−1.043	−.977	−1.020	−1.186
Sign. distributions^a^	0.015	0.00	0.76	0.62	0.34	0.30	0.33	0.31	0.24
Sign. median^a^	0.24	0.05	0.95	0.59	0.45	0.51	0.32	0.29	0.27
*Economics*									
Mean rank (male)	54.60	54.04	49.28	49.16	50.49	50.98	51.42	48.12	48.89
Mean rank (female)	36.09	37.56	50.07	50.39	46.89	45.61	44.44	51.28	51.11
Mann–Whitney *U*	596.5	636	943	934.5	888	853.5	822	883.5	915
Wilcoxon *W*	974.5	1014	3499	3490.5	1266	1231.5	1200	3368.5	3471
*Z*	−2.887	−2.565	−.123	−.191	−.951	−1.012	−1.131	−.495	−.346
Sign. distributions^a^	0.004	0.01	0.90	0.85	0.34	0.31	0.26	0.62	0.73
Sign. median^a^	0.06	0.024	0.65	0.37	0.47	0.32	0.18	0.61	1.0
*Behavioral and educational science*									
Mean rank (male)	28.97	31.24	21.53	21.71	25.35	25.06	23.82	22.24	20.59
Mean rank (female)	19.38	18.00	23.89	23.79	21.57	21.75	22.50	23.46	24.46
Mann–Whitney *U*	136.5	98	213	216	198	203	224	225	197
Wilcoxon *W*	542.5	504	366	369	604	609	630	378	350
*Z*	−2.378	−3.277	−0.585	−0.515	−1.141	−0.847	−0.332	−0.305	−0.960
Sign. distributions^a^	0.017	0.001	0.56	0.61	0.25	0.40	0.74	0.76	0.34
Sign. median^a^	0.01	0.01	0.62	0.62	0.42	0.47	0.91	0.91	0.27
*Psychology*									
Mean rank (male)	54.08	56.61	49.23	49.00	48.43	50.13	48.94	46.13	45.61
Mean rank (female)	38.64	36.61	42.52	42.70	43.16	41.80	42.75	45.00	45.41
Mann–Whitney *U*	657	555.5	851	860	883	815	862.5	975	995.5
Wilcoxon *W*	1932	1830.5	2126	2135	2158	2090	2137.5	2250	2270.5
*Z*	−2.787	−3.609	−1.210	−1.137	−1.187	−1.557	−1.125	−0.203	−0.037
Sign. distributions^a^	0.005	0.00	0.23	0.26	0.24	0.12	0.26	0.84	0.97
Sign. median^a^	0.006	0.001	0.53	0.29	0.36	0.53	0.83	0.83	0.53

Gender = grouping variable; N = 134 (male), N = 109 (female)

See notes Table 1

^a^Two tailed

First for the *whole sample*: As far as (fractionalized) publications are concerned, the median performance and the distribution of performance of women are lower than the median and distribution of the male researchers. However, in all (field normalized) citations-based impact indicators, the differences are small, non-significant, and sometimes women score higher, sometimes men—as Table 4 (part: All) shows. For the *three fields separately*, the pattern is about the same—although in the case of psychology, male researchers have also in the citation-based indicators a somewhat (but non-significant) higher score.

In other words, the null hypothesis that men and women perform equal impact-wise cannot be rejected for most of the indicators. But, we have to reject the hypotheses that productivity is equal among the sexes. Indeed, the productivity gap has increased over the 10 years as becomes visible when comparing Table 1 and Table 3. The Male/Female productivity ratio was 1.09 (mean) and 1 (median), around 2004, and 9 years later the figures were 1.17 (mean) and 1.31 (median). Male researchers now publish on average about 17 % more publications than female researchers, and the former have a 31 % higher median production. For fractional counted publication the increase of the difference is even substantially higher, as is the case for the individual disciplines.^8^

### Performance and careers

**Fig. 1:**
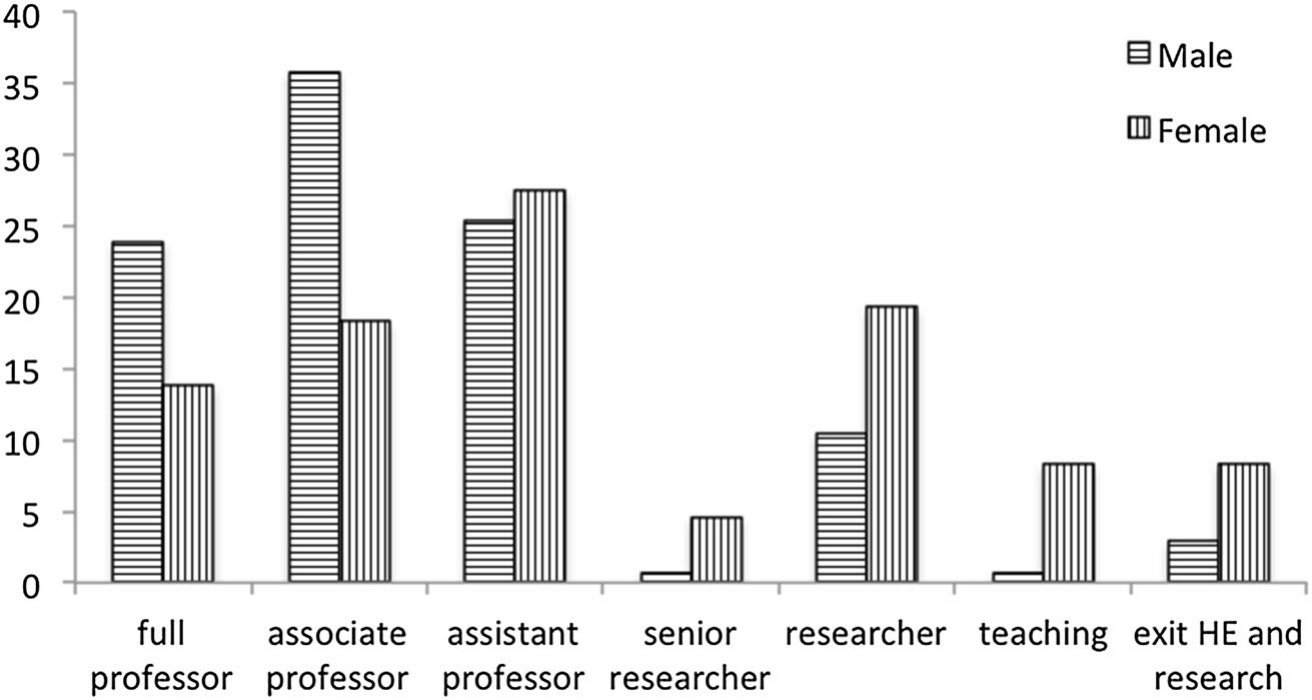
Position by gender

After analyzing the development of male and female research performance, the second question can be addressed: does performance determine academic careers? We collected data about the current position of the researchers in our dataset. Figure 1 shows that gender differences in career are still obvious.

Does performance explain the differences in careers, or gender? In order to test this, we use the SPSS routine *nonparametric tests* to compare the careers of male and female researchers, as the distributions obviously are skewed. The following Table 5 shows the results: The null hypothesis that the career levels of male and female researchers are equal has to be rejected—and the differences are statistically significant (at p = 0.10). In this and the following analyses, we have not included those that left higher education. And because of the small size of the group of senior researchers, we took those together with the class of researchers.

**Table 5:** Average function level by gender

		Average		N
All fields*	Male	13.7	Almost associate professor	125
	Female	12.6	Slightly above assistant professor	98
Economics**	Male	14.0	Associate professor	69
	Female	13.2	Halfway assistant and associate professor	26
Behavioral and educational science**	Male	13.2	Halfway assistant and associate professor	17
	Female	12.2	Slightly above assistant professor	25
Psychology*	Male	13.6	Almost associate professor	39
	Female	12.4	Slightly above assistant professor	47

* Sign < 0.01; ** Sign < 0.10

**Fig. 2:**
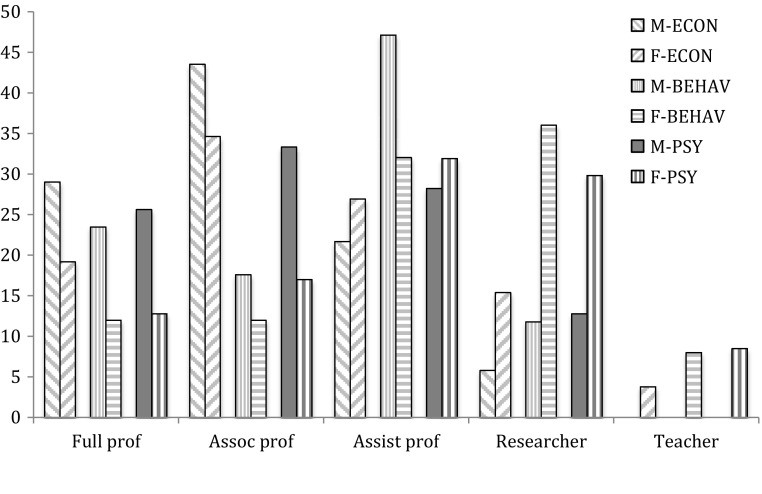
Position by gender by discipline (%)

In terms of careers, male researchers score in average 13.7—almost associate professor. Female researchers score in average 12.6—slightly above assistant professor. Associate professor is also the median position for male applicants, whereas assistant professor is the median for women; so the gender difference is substantial. For the disciplines separately, we find about the same differences, with economics slightly higher and behavioral & education research slightly lower than psychology. Figure 2 shows that the unequal pattern is also visible at the level of the various disciplines separately.

Taking only one variable into account (gender) gives an incomplete picture. In a meritocratic system one would expect that research performance has a strong influence on careers. As the gender differences are in the same (small) order of magnitude for all citation-based indicators, we selected only one impact variable (field normalized citations, with a citation window) for the model, plus productivity. We use fractional counted productivity as we expect panel and committee members to account in some imprecise way for the number of coauthors.

Apart from the performance variables, a few other variables should be taken into account. As we have three academic age groups and relative young researchers, the year of PhD may be a factor: the younger, the less chance to promote to higher positions. The average differences are small—only 1 year—between the groups (2003, 2004, 2005), but as we only consider a 10-years period, a difference of 1 or 2 years is substantial. Another factor may be mobility. If mobility influences the career, and men are more mobile than women, this together may lead to gender differences in careers. We distinguish three levels of mobility: (i) no mobility, (ii) national mobility, and (iii) international mobility. Finally, labor academic markets may de different for the different fields under study. Therefore we also use discipline of the researcher as variable.

We test a model with the researchers’ early 2014 achieved career level as dependent variable, and as independent variables performance (productivity and citations), gender, mobility, academic age, and discipline. Table 6 gives the result of the analysis. For the nominal variables, the effect is tested against one of the conditions: male against female; 2003 and 2004 against 2005; economics and education & behavior against psychology; no mobility and national mobility against international mobility. The fit of the model is good, as the deviance is 555.552 with 879 degrees of freedom.

**Table 6:** Career level by gender, age, mobility and performance

Parameter	B	Std. Error	95 % Wald Confidence Interval		Hypothesis Test			Exp(B)
			Lower	Upper	Wald Chi2	df	Sig.	
Threshold								
Position = 10.00	−1.143	0.5587	−2.238	−0.048	4.185	1	0.041	0.319
Position = 11.00	1.265	0.4748	0.335	2.196	7.099	1	0.008	3.544
Position = 12.00	3.044	0.5075	2.050	4.039	35.983	1	0.000	20.995
Position = 14.00	4.826	0.5600	3.728	5.923	74.264	1	0.000	124.67
Economics versus psychology	1.523	0.3249	0.886	2.160	21.982	1	0.000	4.587
Behavior & edu versus psychology	−0.411	0.3612	−1.119	0.297	1.296	1	0.255	0.663
Year = 2003 versus 2005	0.578	0.3327	−0.075	1.230	3.014	1	0.083	1.782
Year = 2004 versus 2005	0.489	0.2917	−0.082	1.061	2.814	1	0.093	1.631
Mobility: no versus international	−0.073	0.3496	−0.758	0.612	0.044	1	0.835	0.930
Mobility: national vs international	0.080	0.3742	−0.654	0.813	0.046	1	0.831	1.083
FracP	0.305	0.0473	0.212	0.398	41.586	1	0.000	1.357
NCS2y	0.356	0.1682	0.026	0.686	4.471	1	0.034	1.427
Male versus female	0.549	0.2804	0.000	1.099	3.835	1	0.050	1.732
Scale	1^a^							

Ordinal response (multinomial) model with a cumulative logit link function

Dependent Variable: achieved rank early 2014

Model: (Threshold), FracP, NCSf2y, year, gender, discipline, mobility

Model fit: deviance = 555.552, df = 879

^a^Fixed at the displayed value

First of all, the labor market seems better and careers seem faster in economics than in psychology, and in psychology better than in education & behavior.

Secondly, the year group variable has an effect on the achieved career level: if one belongs to the older cohorts 2003 and 2004, the average achieved career level is higher than for those in the cohort 2005. This is of course expected: the longer the career, the more time one has had for moving up.

Thirdly, mobility has only a small (and non-significant) effect. Note that the small effect does follow what would be expected: international mobility is slightly better than national mobility, which is slightly better than no mobility.

Fourthly, scholarly performance has a statistically significant positive effect on the career.

Finally, the analysis shows that gender has an effect on achieved career level as male researchers have achieved a higher average career level than female researchers, when controlling for all other variables.

## Conclusions and discussion

Quite some research literature focuses on gender bias in career and grant decisions. Unfortunately most of these studies do not control for possible performance differences, which makes conclusions about gender bias impossible. In this paper we provide an alternative to this practice as we combine data on performance with data on careers and mobility. Our sample consists of researchers who around 10 years ago were all about 3 years after their PhD, and as Table 1 and 2 show, female and male researchers had on average about the same performance. Using better data and more sophisticated indicators than the earlier paper (Van Arensbergen et al. 2012) we come to the same conclusion. The analysis confirms that gender performance differences in the early career may be disappearing, a result that sharply contrasts to what has been concluded from earlier studies.

The dataset enabled us to investigate whether gender differences in performance and career have developed over time. The average impact of papers of male and female researchers remained about equal. But in a period of some 10 years, the productivity of the male researchers has become higher than of female researchers. This divergence suggests that we observe a change in the academic life cycle (differences emerge later) than a generational effect (no differences any more in the current generation).

In a second step, we analyzed the development of careers, and we find that male researchers had a much better career in the first about 13 years after their PhD than female researchers. In our sample, about 61 % of the male researchers became full or associate professor within this period. For female researchers this was half of that: 32 %. When also taking into account academic age, performance, mobility and discipline, the picture remains the same: gender has considerable effect on the achieved career level. Obviously, gender bias seems to prevail in academic hiring, as the differences in career development cannot be explained in terms of performance indicators only—next to objective differences such as cohort and discipline.

The most burning question concerns the mechanisms underlying performance and career differences. Why these differences in productivity and career have emerged remains an issue for further research. However, several possibilities can be discerned.

1. Mobility had no significant effect on careers. This may change if one takes the *quality* of the mobility into account, by distinguishing the affiliations in terms of the ranking of the institution (e.g., in the Leiden Ranking), and distinguishing between upward and downward mobility.
2. We also showed that differences in career partly are an effect of gender bias. This may be the effect of organizational processes and procedures, which were not under study in this paper. But they remain an important research topic.
3. The observed productivity differences may be spurious and an effect of different topic choice within the disciplines. By using *field*-*adjusted productivity indicators* (Sandström and Wold 2015) one may be able to test this, at disaggregated level. Together with other improvements such as the deployment of size-dependent indicators^9^ (Van den Besselaar & Sandström 2015), we will further investigate this in a coming article.
4. Another possible explanation for gender differences in research productivity relates to marriage and family building. However, the research literature is quite consistent: Several studies show that the academic career of female scientists is not necessarily affected negatively by parenthood (Fox & Faver 1985; Cole & Zuckerman 1991; Astin & Bayer 1979; Dryler 2011), and therefore we wouldn’t search in this direction for explanations. Some analyses indicate that for specific academic positions, e.g. assistant professor, there is a slight advantage for women to have children of school age (Dryler 2011). One should be aware that these findings come from studies mainly covering the US and the Nordic countries. Despite the fact that all other circumstances are about the same, i.e. modern, highly developed nations with strong science systems, the situation in the country under study (the Netherlands) may be different and could be further investigated.
5. Alternatively, there are good arguments to search for explanations in the area of self-selection. Men and women tend to leave the academic career for other options due to different reasons, among them structural discrimination on the labor market. Gender differences can roughly be described in terms of men searching for a new career and higher pay, while women move away from academia because they are dissatisfied with their working conditions and more seldom find an alternative career (Dryler 2011). As a consequence, lower performing men may leave academia for another career more often than lower performing female researchers who may more often continue their academic career. This kind of patterns may explain our findings.
6. A still not very much explored set of models relates to team collaboration and co-author relations, which could moderate the effect between gender and performance (Badar et al., 2014; Verbree et al., 2015).
7. We showed that differences in career partly are an effect of gender bias. Apart from that, performance differences play a role. However, the growing gender difference in productivity may itself be an effect of bias. It could be well the case that in the beginning equally performing men and women enter—through gender stereotyping and biased decisions—into diverging early career trajectories (e.g., lower and teaching oriented positions versus higher and more research oriented positions) which in turn leads to increasing productivity differences. And these increased performance differences together with existing gender bias may reinforce the career differences between male and female researchers. These possible explanations are as many hypotheses for further research.

## Footnotes

1 This may be the strongest policy effect of studies on gender bias: such studies focus attention on the problem, and force councils to act and to try to solve it. Despite these efforts, the causal mechanisms under gender bias are not taken away. Consequently, without permanent attention gender bias can be expected to creep into the system again.

2 This section is based on Van Arensbergen, et al. (2012).

3 Although there is evidence suggesting that the effect of marital status is less univocal (Fox 2005).

4 The processes described in this paragraph may not take place everywhere and also not in the same way or speed.

5 For most applicants we have a publication list obtained from their own website or the universities’ website. However, researchers differ in what they include in that list. So these lists can only be used to correct the retrieved publications, and not as a reliable source as such.

6 One of these years depending on the researcher: the data come from career grant applications over a three years period. As the applications have to be done within three years after obtaining the PhD, the year reflects the academic age. One or two years longer may influence the overall performance until 2014, and also the career steps made.

7 In (Van Arensbergen et al. 2012) the data were based on *automatic* coupling of application data with Social Sciences Citation Index publication data with a Dutch address, using family name and first initial. This procedure accepts some error based on homonyms and synonyms. Additionally, some applicants use different first initials in applications compared to publications, and in some cases the academic titles were taken for first names (for the last point: Van den Besselaar et al. 2014). We recollected all data *manually* in order to avoid these problems. In contrast to the previous version, we also extended the search, as we now also included non-Dutch addresses and Science Citation Index-expanded publications. Finally, we now use an equal citation window (up to 31-12-2006; and a 2-years citation window). The results of the analyses and the conclusions remain highly similar, suggesting that the differences even out. The main difference is that in (Van Arensbergen et al. 2012) we found that women seem to outperform men in the *top* of the distribution. The new and better data and analysis suggest equal performance (see the *findings* section below).

8 Table 1 and Table 3 also report the figures for the individual disciplines. For economics, the men were on average 28 % more productive in the initial measurement, and ten years later this has increased to 50 %. For psychology, the corresponding figures are 21 % and 59 %.

9 Size-dependent indicators measure e.g. the *absolute number* of top papers, and not the *share* of top papers within the oeuvre of a researcher.
